# Notes and Formulæ

**Published:** 1889-07

**Authors:** 


### PRACTICAL NOTES AND FORMULÆ.

**Thompson's Fever Syrup.**—Dr. C. S. Harris of Rome Ga., writes: "I send you the formula of the famous *Fever Syrup*, so popular thirty or forty years ago as a remedy in typhoid and continued fevers, and which, in my own long experience in the profession, has proven the most efficient remedy in typhoid fever I have ever known. I yet use it with remarkable success:

| | | |
|---|---|---|
| ℞ | Syrup of rhubarb | 4 ounces. |
| | Tinc. valerian | 2 ounces. |
| | Oil of sasafras | 20 drops. |
| | Piperin | 10 grains. |
| | Bi. carb. soda | 20 grains. |

Mix the oil of sassafras and piperin strongly with the syrup before adding the valerian. Dose, from a teaspoonful to a tablespoonful, in four or five tablespoonfuls of sweet milk, every three hours.

Sometimes, though not often, I give quinine in connection with the syrup. The syrup is given both during the exacerbation of fever and the intermission.

In small children I reduce the quantity of piperin in the formula to 5 grains.

**Ointment fer Piles.**—The following ointment is soothing and curative in piles:

| | | |
|---|---|---|
| ℞ | Ung. stramon | oz. j. |
| | Ung. gallæ | oz. j. |
| | Cerat. plumbi subacetates | oz. j. |
| | Pulv. opii | dr. ss. M. |

—*Ind. Med. Rep.*

**Diphtheria.**—Stellded, a Swedish doctor, has used the following in 1400 cases of diphtheria, since 1882, with only 69 deaths:

| | | |
|---|---|---|
| ℞ | Cyanide Mercury | ½ grain. |
| | Tinct. aconite | ½ drachm. |
| | Honey | 3 ounces. |

M. Sig.: A teasponful every fifteen, thirty or sixty minutes, according to the age of the patient.—*Med. Brief.*

**Orchitis.**—

| | | |
|---|---|---|
| ℞ | Ammonii muriatis | 2 drachms. |
| | Spts. Vini Rect. | 2 ounces. |
| | Listerine | 2 ounces. |

M. Sig.: Apply constantly to inflamed testicle.

**Tape Worm.**—

| | | |
|---|---|---|
| ℞ | Chloroformi | 1 drachm. |
| | Glycerini | 3 drachms. |
| | Ol. Tigili | 1 drop. |

M. Sig.: All at one dose, on an empty stomach, after fasting twenty-four hours.—Dr. J. W. Freeman, in *Med. Brief.*

**For Diarrhœa of Children.**—

| | | |
|---|---|---|
| ℞ | Bismuthi subnit | ʒ iss. |
| | Lime water | fl. ʒiii. |
| | Syr. aurant flor. | fl. ℥ i. |
| | Aquae fl, feniculi q. s. | ad. ℥v. |

M. Teaspoonful every two hours.

**Anti Emetic.**—

| | | |
|---|---|---|
| ℞ | Creosote | 20 drops. |
| | Acetic acid | 40 drops. |
| | Sulp. morph. | 2 grs. |
| | Water | 2 onces, |

M. Dose, teaspoonful in nausea and vomiting repeated in half hour if necessary.

**Gray Oil.**—E. Lang (quoted in the B. M. J., June 16th, 1888, from a paper read by him before Soc. of Physicians, Vienna), uses the following formula for hypodermic injections in syphilis:

Hydrarg.
Lanolin, ää 3 parts.
Ol. oliv. 4 parts.
M. Sig. For hypodermic use.

This contains 30 per cent. of mercury. Lang found that an injection of 0.3 of the oil for one week was sufficient to cause most of the symptoms to disappear.—Dr. Pratt, in *Maryland Med. Jour.*

**Diphtheria.**—Listerine alone, in combination with other drugs or antiseptics, may be used to spray the throat and fauces, and to diminish the dangers of septic absorption.

| | | |
|---|---|---|
| ℞ | Listerine | ℥i. |
| | Water | ℥vij. |

M. Sig. Spray and gargle frequently and allow patient to swallow freely.

| | | |
|---|---|---|
| ℞ | Listerine | ℥ss. |
| | Glycerine | ℥ss. |
| | Water | ℥iij. |

M. Sig. Spray every two hours.

| | | |
|---|---|---|
| ℞ | Listerine | |
| | Solution cholorinated | |
| | Soda ää | ℥ss. |
| | Water | ℥iij. |

M. Sig. Spray every two or three hours.

Its employment with tannic acid has been especially recommended in a wide range of diseases.—*Ex.*

